# A Case of Peritoneal Dialysis–Associated Peritonitis Due to *Mycobacterium tuberculosis*: Case Report and Review of the Literature

**DOI:** 10.1155/crdi/5448802

**Published:** 2026-07-10

**Authors:** Jenna Maco Wick, Joseph David Cooper

**Affiliations:** ^1^ Division of Infectious Diseases and Geographic Medicine, Stanford University, Stanford, California, USA, stanford.edu; ^2^ Division of AIDS Medicine and Infectious Diseases, Santa Clara Valley Healthcare, San José, California, USA

**Keywords:** end-stage renal disease, peritonitis, tuberculosis

## Abstract

A 73‐year‐old man from the Philippines with Type 2 diabetes mellitus and end‐stage renal disease on peritoneal dialysis developed peritonitis. The infection was refractory to empiric therapy, and the pathogen was identified as *Mycobacterium tuberculosis*, when 4 weeks after collection, a preliminary positive AFB culture underwent PCR testing. He did not initially tolerate a regimen of isoniazid, rifampin, pyrazinamide, and ethambutol but temporarily tolerated rifabutin, levofloxacin, and ethambutol and ultimately tolerated a regimen of levofloxacin, ethambutol, and pyrazinamide. This case highlights the diagnostic and treatment challenges of peritonitis due to tuberculosis, emphasizing the importance of including tuberculosis in the differential diagnosis. Given the severity and the limited current understanding, further research is needed to guide effective management and improve outcomes.

## 1. Introduction

Tuberculosis remains a significant public health threat, with an estimated 10.8 million people developing tuberculosis disease in 2023 [1]. Patients with end‐stage renal disease are at a significantly higher risk for tuberculosis, with an incidence ranging from six to 25 times greater than the general population [2], which is due to the impaired cellular immunity as well as demographic characteristics [3]. Patients on peritoneal dialysis, in particular, face an increased risk of peritonitis due to tuberculosis, which, though a rare presentation of tuberculosis overall, occurs in this patient population nearly as frequently (37%) as pulmonary tuberculosis (40%) [4]. This is due to impaired phagocytic and lymphocytic activity in the peritoneal cavity because of the lower pH, higher osmolality, and greater fluid volume caused by the peritoneal dialysate [5]. Peritonitis due to tuberculosis can be challenging to diagnose and treat as seen in this case, and we review the current practices and areas in need of greater knowledge.

## 2. Case Presentation

**FIGURE 1:**
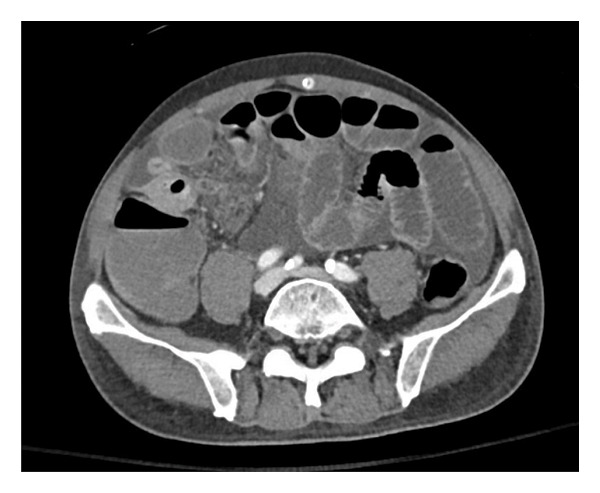
CT of abdomen and pelvis without contrast.

Here, we present a case of peritonitis in a 73‐year‐old man who immigrated from the Philippines 23 years ago, with Type 2 diabetes mellitus (hemoglobin A1c 8%) and end‐stage renal disease on continuous ambulatory peritoneal dialysis for the past six years. He developed cloudy peritoneal drain fluid, postprandial abdominal pain, weakness, and weight loss 3 weeks prior to presenting to an outpatient nephrology clinic. He was afebrile, with no systemic leukocytosis. Peritoneal dialysate fluid studies revealed 150 white blood cells/μL with 17% neutrophils and 42% lymphocytes; aerobic bacterial and fungal cultures were negative. He was empirically started on intraperitoneal ceftazidime. However, repeat peritoneal dialysate fluid studies after four days revealed an increase in white blood cells to 627 cells/μL, and he was admitted to the hospital. Abdominal exam was notable for only mild distension without tenderness to palpation, and CT of the abdomen and pelvis showed a large volume of ascites with mesenteric and omental edema (Figure 1). He was started on intraperitoneal vancomycin and ceftazidime for three weeks, and additional oral levofloxacin 250 milligrams every 48 h was added for the last 11 days due to ongoing uncertainty in his case. Despite completing this treatment regimen, he did not have significant improvement in symptoms. Further diagnostic testing with 16 S rRNA bacterial sequencing of the dialysate fluid resulted as negative. Twenty‐eight days after the initial peritoneal dialysate fluid sample was collected, the culture was positive by AFB/Kinyoun stain, and subsequent PCR testing was positive for *Mycobacterium tuberculosis*. The culture finalized as *M. tuberculosis*, susceptible to rifampin, isoniazid, ethambutol, and streptomycin. The patient had no known exposure to tuberculosis and no prior history of tuberculosis diagnosis or treatment. An interferon‐gamma release assay (QuantiFERON) performed 3 years prior was negative.

The patient was transitioned to hemodialysis, and the decision was made to remove the peritoneal dialysis catheter. Three days after diagnosis of tuberculosis peritonitis, the patient underwent peritoneal dialysis catheter removal by general surgery without complications. Although he had no respiratory symptoms, chest CT revealed multiple solid pulmonary nodules, and he was evaluated for pulmonary tuberculosis, with three AFB sputum cultures and two sputum *M. tuberculosis* PCR tests that were all negative.

Rifampin, isoniazid, pyrazinamide, and ethambutol were initiated and adjusted to his renal function. Serum rifampin and isoniazid two‐ and six‐hour levels were collected and were within the normal range, showing slow adequate isoniazid absorption (Table 1). However, he developed elevated liver function tests (total bilirubin 4.8 mg/dL, direct bilirubin 3.8 mg/dL, aspartate aminotransferase 209 U/L, alanine aminotransferase 18 U/L, and alkaline phosphatase 169 U/L), along with worsening postprandial abdominal pain and antituberculosis therapy was held after 12 days of treatment out of concern for an adverse medication effect. Tuberculosis hepatitis was also considered but deemed unlikely when liver function tests improved after 7 days of holding medications. He was rechallenged with a regimen considered to be less hepatotoxic and initially tolerated rifabutin, levofloxacin, and ethambutol, with resolution of abdominal pain, loss of appetite, and weakness. However, he then developed thrombocytopenia (71 cells/μL), and rifabutin was stopped. Pyrazinamide was restarted, and the regimen of levofloxacin, ethambutol, and pyrazinamide was well tolerated with a plan to continue for a total 9‐month treatment duration due to the nonstandard regimen.

**TABLE 1:** Therapeutic drug‐monitoring levels.

	Patient’s plasma concentration 2‐h level	Patient’s plasma concentration 6‐h level	Normal plasma concentration range
Rifampin	16.77 mcg/mL	10.32 mcg/mL	8‐24 mcg/mL
Isoniazid	1.94 mcg/mL	3.35 mcg/mL	3‐5 mcg/mL

## 3. Discussion

Peritonitis due to tuberculosis has significant complications including a mortality ranging from 18% to 58.3%, with diabetes mellitus as a significant risk factor for increased mortality [6], which warrants further research to better understand and potentially mitigate. In addition to severity, it is a challenging entity in many aspects of diagnosis and management. Although characteristically there will be an elevated white blood cell count in the dialysis effluent, there have been reported cases where it is lower than threshold of 100 cells/μL [6], and patients should undergo further workup if there is clinical suspicion. Most cases of peritonitis due to tuberculosis have a neutrophilic predominant dialysate fluid white blood cell count [7], but lymphocytic predominance has also been seen as it was with our patient. Diagnosis can be delayed due to the slow nature of AFB culture, and for our patient, it took 4 weeks from peritoneal dialysate fluid collection to initial AFB culture positivity. Delayed diagnosis can lead to significant delays in treatment, with studies showing a mean treatment delay of 15–77 days [6]. Many patients are initially misdiagnosed with bacterial peritonitis, with one study reporting that this occurred in 68% of cases [7] and fail empiric treatment prior to diagnosis as with our patient. Accurate diagnosis is challenging as peritoneal fluid AFB smears have a sensitivity of less than 20% [8], and although peritoneal fluid AFB cultures have an increased sensitivity of 70%–90%, they take at least 10–14 days or more to become positive [8]. Peritoneal biopsy cultures offer greater sensitivity of 90% [9], but performing a biopsy is invasive with associated risks. *M. tuberculosis* PCR testing has been shown to have a sensitivity of 50%–85.7% for peritonitis due to tuberculosis and is associated with a shorter time to treatment initiation and shorter length of hospitalization [10]. Rapid diagnostics with *M. tuberculosis* PCR testing should be considered in patients with risk factors to decrease delays in diagnosis and treatment.

There is uncertainty regarding whether it is necessary to remove the peritoneal dialysis catheter; data are conflicting with some studies showing an association with improved mortality [11] and others finding no significant benefit [7]. The International Society for Peritoneal Dialysis guidelines give a 2C recommendation (suggestion based on low quality of evidence) to treat with antituberculosis drugs without catheter removal as first‐line therapy [12]. Antituberculosis treatment may be problematic; notably, rifampin has decreased bioavailability in dialysate fluid [13], and therapeutic drug monitoring of serum should be considered to guide adequate dosing [14]. Although peritoneal drug levels are done in a research setting, it is not standard in clinical practice in part because the literature and guidelines focus on serum therapeutic drug monitoring. Patients with end‐stage renal disease appear to have a trend toward increased adverse effects with antituberculosis medications [15], as was observed with our patient; however, the mechanism and significance are unknown, and further information is needed. Peritonitis due to tuberculosis is a rare but severe condition that presents significant diagnostic and therapeutic challenges, and more research is needed to establish optimal management protocols and improve patient outcomes.

## Funding

No funding was received for this manuscript.

## Conflicts of Interest

The authors declare no conflicts of interest.

## Data Availability

Data sharing is not applicable to this article as no datasets were generated or analyzed during the current study.
